# Oral Submucous Fibrosis and Scleroderma: A Review of the Etiopathogenesis, Clinicopathological Correlation, and Management Aspects

**DOI:** 10.7759/cureus.44502

**Published:** 2023-08-31

**Authors:** Sreedevi J, Lubnaz S, Maneesha V Nair, Karuna Thulasi R, Priya Ramani

**Affiliations:** 1 Department of Oral Medicine and Radiology, Thai Moogambigai Dental College and Hospital, Chennai, IND

**Keywords:** scleroderma, submucous fibrosis, oral premalignancy, inflammation, hyalinization, collagen disease, autoimmune, antibodies

## Abstract

Oral submucous fibrosis (OSMF) is a chronic, progressive, insidious premalignant disease with multifactorial etiology affecting any part of the oral cavity and sometimes the pharynx by triggering a rapid onset of trismus and dysphagia due to stiffness at the lips, cheek, pharynx, and upper oesophageal region. Submucous fibrosis resembles many auto-immune, dermatological, mucocutaneous, and fibrotic lesions that include scleroderma, amyloidosis, iron deficiency anemia, and systemic or generalized fibromatosis clinically and histologically. Several authors established an association between oral submucous fibrosis and scleroderma with predominant oral manifestations on the basis of similarity in clinical and histological characteristics despite different pathogenesis and prognostic aspects. Scleroderma or systemic sclerosis is an autoimmune connective tissue disorder clinically manifested as fibrosis of the skin, blood vessels, and visceral organs with or without the involvement of the oral cavity. Thus, understanding the disease mechanism, appropriate early diagnosis, and clinical management of these two entities play an important role in disease prognosis and treatment outcomes. The present review was carried out to briefly present a concise overview of the etiopathogenesis, clinical, histological, diagnosis, and management aspects of OSMF and scleroderma based on the available literature, with special emphasis on similarities and differences between these two entities subsequently aiding in appropriate treatment planning.

## Introduction and background

Collagen is a major structural protein that represents the primary fibrous component of the extracellular matrix including skin, muscle, tendon, bone, and cartilage. The synthesis and degradation of collagen is an active process and any alterations in the collagen structure or mechanism will affect the oral tissues. The common connective tissue disorders with characteristic oral manifestations are systemic lupus erythematosus (SLE), systemic sclerosis/scleroderma (SSc), rheumatoid arthritis (RA), and Sjögren syndrome (SS), which often impose challenges in diagnosis and treatment owing to their uncertain presentation and unpredictable prognosis [1-3]. Several authors have established an association between SSc and oral submucous fibrosis (OSMF) based on a similarity in clinical and histological characteristics despite different pathogenesis and prognostic aspects [4-6].

OSMF is an insidious, progressive, long-standing disease affecting any part of the oral cavity. Sometimes, the pharynx is often associated with a juxta-epithelial inflammatory reaction followed by a fibroelastic transformation of the lamina propria and epithelial atrophy histopathologically, preceded by vesicle formation, thus triggering the rapid onset of trismus, severe pain, and dysphagia due to stiffness in the cheeks, lips, perioral region, pharynx, and upper esophageal region. It is a chronic, pre-cancerous condition commonly seen among middle-aged groups with higher prevalence in the Southeast Asian subcontinent, including India. Although etiopathogenesis is not fully established, a multifactorial etiology is proposed, with areca nut as the chief causative agent while smokeless tobacco, chilies, micronutrient deficiencies, vitamin deficiencies, malnutrition, toxic copper levels in food products, autoimmunity, and genetic predisposition are often considered contributing risk factors [7-9].

Scleroderma (SSc) is an uncommon connective tissue disorder affecting the skin and internal organs with a wide array of oral manifestations predominantly associated with fibrotic manifestations and widening of the periodontal ligament space, and neurological systems often resemble OSMF, which was previously termed idiopathic scleroderma of the mouth although SSc primarily includes sclerosis of the skin and other extremities rather than the oral counterpart. OSMF is believed to be a localized collagen disease of the oral cavity, whereas scleroderma or systemic sclerosis is a heterogeneous collagen disorder characterized by thickening of the skin, extensive fibrosis, abnormal nail fold capillaries, and vasculopathy because of increased deposition of the extracellular matrix caused by fibroblast dysfunction with excessive production of autoantibodies. The aetiopathogenesis of SSc is not fully established; however, antibodies directed against the epithelium, with a strong association with SSc-specific autoantibodies, and cell-mediated immunity were suggested based on immunological studies [10-13].

The differential diagnosis of scleroderma with an oral manifestation is ascertained as OSMF, which challenges the clinicians due to similar clinical and histopathological findings. The review was carried out to briefly present a concise overview of the etiopathogenesis, clinical, histological, diagnosis, and management aspects of OSMF and scleroderma based on the available literature, with special emphasis on similarities and differences between these two entities subsequently aiding in appropriate treatment planning.

Literature overview

Our review of the literature included the following findings: 1. Desa JV (1957) revealed that scleroderma preliminarily affected the skin (cutaneous) with occasional oral mucosa manifestations while OSMF affects the oro-pharyngeal regions with no established cutaneous or visceral spread [14]; 2. Binnie WH and Cawson RA (1972) concluded a definitive lack of evidence of a connection between OSMF and progressive systemic sclerosis despite similar clinical and histological features likely due to the sharing of a defect of collagen maturation [15]; 3. Morawetz et al (1987) observed a similarity between OSMF and scleroderma clinically and histopathologically; however, they detected that OSMF is not characterized by serological changes as found in scleroderma [16]; 4. Wood RE and Lee P (1988) demonstrated that the oral manifestations of systemic sclerosis appeared as a characteristic “purse string” furrow and a widening of the periodontal ligament space [17]; 5. Rajendran R (1994) suggested that the major histocompatibility complex (MHC)-mediated immunological instability is effective in both disease entities [7]; 6. Rao NR et al. (2020) proposed an inter-professional approach for the early diagnosis and appropriate management of both oral and systemic symptoms of OSMF, to reduce mortality and morbidity rates [6].

## Review

Methodology

A structured literature search for articles written in the English language in PubMed/MEDLINE, EBSCOhost, Google Scholar, Scopus, IEEE Xplore Digital Library, and Web of Science databases was conducted by using the following MeSH terms: “Oral Submucous Fibrosis” OR Oral Scleroderma” AND “Dental”, “Systemic Sclerosis” AND “Autoimmune diseases” "Collagen disorders, Oral" OR “Premalignant Conditions” OR “Connective tissue” OR "All Metadata", “Oral Manifestations, Dermatology”, “Fibrosis, Mucocutaneous”, “Oral Precancer, Arecoline”, “Histopathology, OSMF” AND “Oro-facial manifestations, Scleroderma”.

Epidemiological considerations

OSMF is one of the most common premalignant conditions of the oral cavity, affecting more than five million people globally, and is predominantly seen among the South and Southeast Asian subcontinent population with a prevalence of 0.2-2.3% in males and 1.2-4.6% in females. It is common among 11 to 60-year-olds in India, presenting with about 2.3-7.6% malignant transformation depending on diagnostic criteria and follow-up interval [1,2,7-9].

Steen V et al., Weeding et al., and Hill et al. showed an increased prevalence of SSc among Blacks (race and ethnic predilection), with an annual estimated incidence of 19% per million per year and an overall prevalence of 250 per million approximately, and globally predominantly seen among females compared to males (1:3), with wide-ranging ages with peaks from 30 to 50 years [18-20]. Morphea, a plaque form of localized scleroderma affecting mostly women with an incidence of three cases per 100,000 individuals every year, is more prevalent in adults while linear scleroderma affects mostly children less than 12 years of age [21].

Khan AM et al., Jain A et al., Kumar KK et al., and Hazarey VK et al., in their epidemiological studies, observed higher OSMF instances among the young to middle-aged groups and school-going children, with a higher male predilection owing to habit addiction [22-25].

Oral submucous fibrosis: etiology, risk factors, and etiopathogenesis

**Figure 1:**
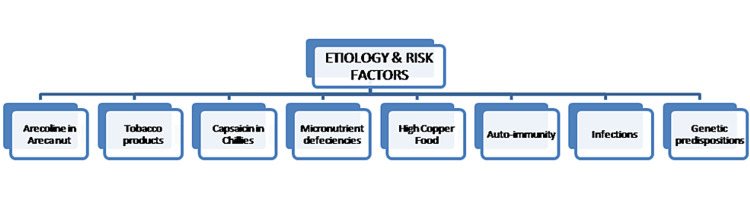
Etiology and risk factors of oral submucous fibrosis Image Credit: Dr. Sreedevi J

**Figure 2:**
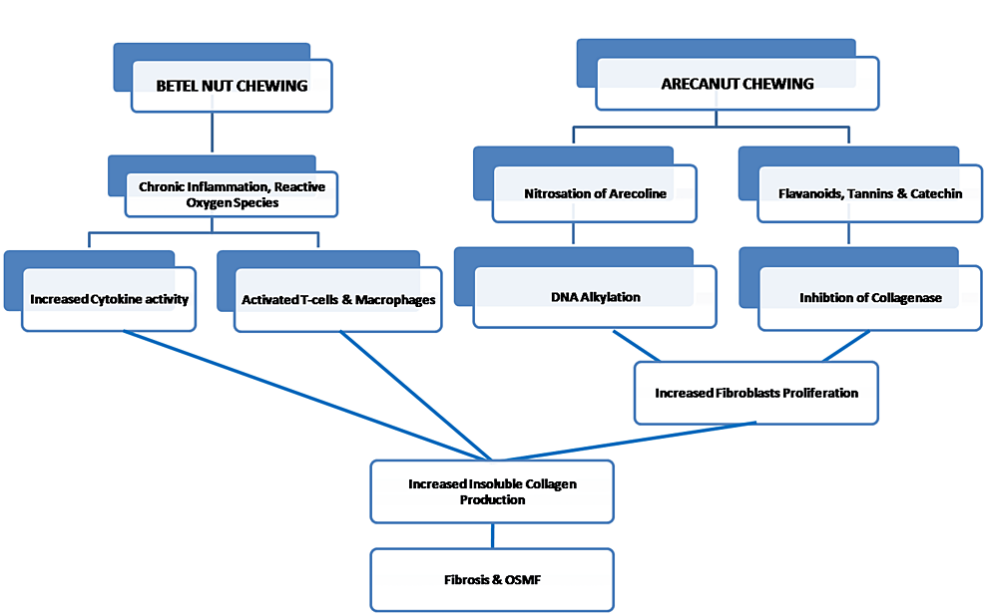
Etiopathogenesis of OSMF Image Credit: Dr. Sreedevi J OSMF: oral submucous fibrosis

Figures 1, 2 show the etiology, risk factors, and etiopathogenesis of OSMF.

Scleroderma: etiology, risk factors, and etiopathogenesis

**Figure 3:**
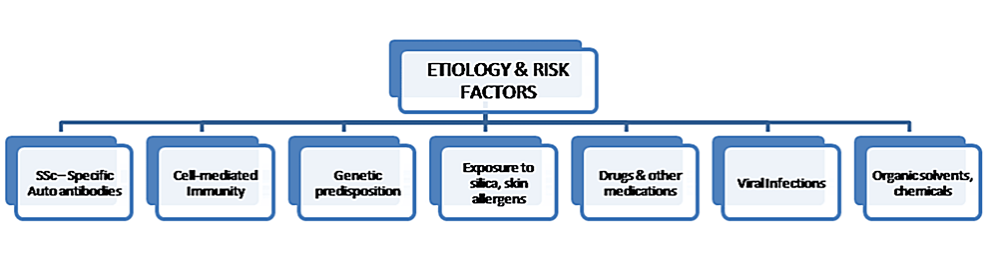
Etiology and risk factors of scleroderma Image Credit: Dr. Sreedevi J

**Figure 4:**
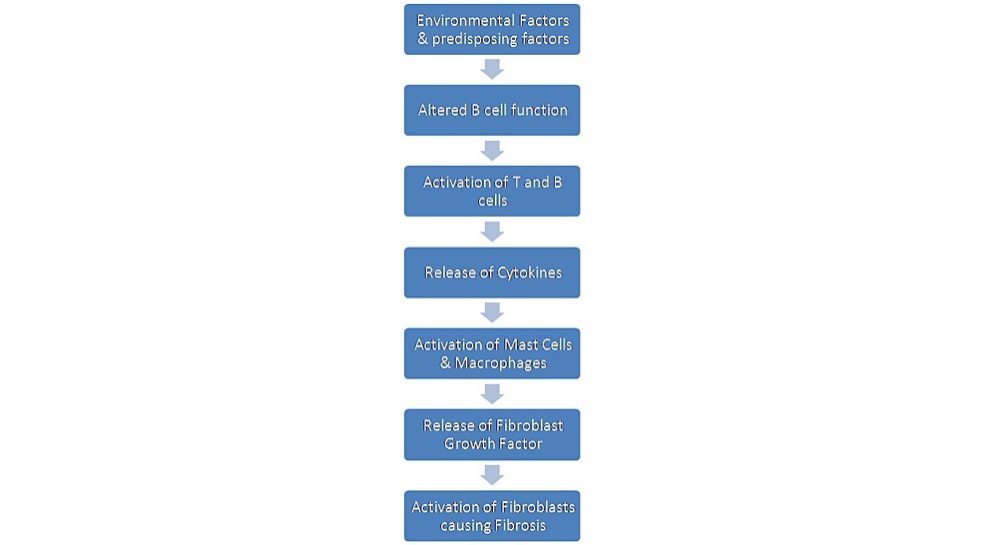
Etiopathogenesis of scleroderma Image Credit: Dr. Sreedevi J

Figures 3, 4 show the etiology, risk factors, and etiopathogenesis of scleroderma.

Clinical features of OSMF and scleroderma

Table 1 shows the clinical features of OSMF and scleroderma [26].

**Table 1:** Clinical features of OSMF and scleroderma OSMF: oral submucous fibrosis; TMJ: temporomandibular joint

	OSMF	SCLERODERMA
INTRAORAL FEATURES		
Oral mucosa	Diffuse blanching (marble stone)	Pale and firm
Pigmentation	Less	Less
Papilla	Less	-
Uvula	Shrunken (inverted hockey stick appearance)	-
Erosion and ulceration	Seen	-
Tongue	Restrictive movements	Stiff
Periodontal ligament space widening	Absent	Present
Resorption of alveolar bone	Absent	Present
Epithelial atrophy	Present	-
Salivation	Reduced	Reduced
Buccal mucosa	Thick fibrous bands	-
Telangiectasia	-	Present
Teeth	Missing	Increased decay and missing
Gingival recession	Absent	Present
Capillaroscopy	-	Detection of abnormal nail fold capillaries
EXTRAORAL FEATURES		
Mouth opening	Reduced	Reduced
Lips	Thinning	Retraction
Cheeks	Sunken with multiple folds	-
Deglutition	Difficulty experienced	-
Weight loss	Extreme	-
Atrophy of musculature	Present	-
Ankylosis of TMJ	-	Present
Dysphagia	Present	Present
Hard and soft palate	Fibrosis	Fibrosis

Histopathological features of OSMF and SSc

Table 2 shows the histopathological features of OSMF and SSc [1-5,27-34]

**Table 2:** Histopathological features of OSMF and SSc OSMF: oral submucous fibrosis; SSc: scleroderma

	OSMF	SSc: associated oral manifestation features
Characteristic Findings	1. Sub-epithelial inflammatory reaction	1. Perivascular inflammatory infiltrate
	2. Juxta-epithelial hyalinization degenerative and atrophic changes in muscular layer fibrosis	2. Increased extracellular matrix, thickened blood vessels, loss of dermal papillary structures, fibrosis
Histological Stages	Stages - inflammation, hyalinization, fibrosis, malignant transformation	Stages - inflammation, vasculopathy, fibrosis/sclerosis
Epithelium	Atrophic epithelium showing dysplastic changes at the later stages with loss of rete ridges	Normal or atrophic/flattened superficial layer with loss of rete ridges. Nodular collection of lymphocytes at the dermal-subcutaneous junction
Connective tissue stroma	1. Granulation, degenerative, and atrophic changes in the muscular layer. 2. Fibrosis - complete collagen hyalinization, obliterated blood vessels, lymphocytes, and plasma cells, dense band of collagen bundles. 3. Malignant transformation - dysplasia, erythroplakia, and changes into squamous cell carcinoma	1. Vasculopathy - edematous endothelial cells Submucosal hyalinization with thickening of blood vessels. Later stages show the disappearance of subcutaneous fat and severe sclerosis at the walls (intima) of the blood vessels. 2. Fibrosis/Sclerosis-thickening& hyalinization of the collagen fibers in the skin. 3. Deposition of collagen fibers (sclerosis) at the reticular layer. 4. Atrophy, degeneration, and drop out of adnexal structures “entrapped” by excessive collagen deposition

Laboratory diagnosis

Table 3 lists the laboratory diagnosis.

**Table 3:** Laboratory diagnosis

	Oral submucous fibrosis	Scleroderma
Hemoglobin	Decreased	Normal
Serum Iron	Decreased	Normal
Serum protein	Decreased	Normal
Vitamin B 12 complex	Decreased	Normal
Folic acid	Decreased	Normal
ESR	Increased	Increased
Cu, Zn, Albumin, Mucoproteins	Decreased	Normal
T lymphocyte count	Decreased	Normal
Eosinophils	Normal	Decreased

Immunological markers

Serological analysis in OSMF shows increased serum levels of IgA, IgD, IgE, Beta-2-microglobulin, and HLA typing (A10, B7, and DR3) [2,6-9]. Many immunochemistry studies have revealed CK2, CD3, and CD4-positive cells at the epithelial, juxta-epithelial, and basilar regions. Altered expression of tumor suppressor and promoter genes (p53, bcl-2, bax, and ki-67), mutations in the adenomatous polyposis coli gene along with an increased level of vimentin, plasminogen activator inhibitor-1, Heat Shock Protein (HSP-47), keratinocyte growth factor-1, and cystatin C in OSMF patients also indicates the increased risk of carcinomatous changes [2,7-9].

Clinical chemical investigation of SSc includes transaminase (alanine transaminase - ALT, aspartate aminotransferase - AST), cholestasis parameters (λ-GT, AP), lactate dehydrogenase (LDH), creatinine, creatinine kinase (CK), and aldolase. In advanced stages, elevated levels of creatinine >10% indicate renal impairment with multiple organ involvement.

Cytological studies

Cytogenetic studies on OSMF revealed increased AgNOR (silver binding nucleolar organizer region) proliferative activity, and salivary marker studies revealed higher levels of S-100A7, peroxidases, lactic acid dehydrogenases, and 2-hydroxy 8-deoxy-guanosine with decreased superoxide dismutase, vitamins, antioxidants, and micronutrients in saliva indicating the malignant risk potential of the disease.

SSc-specific autoantibody Scl-70, anti-topoisomerase 1, or ACA (anti-centromere antibody) are considered the primary serological marker in SSc. The presence of systemic disease markers in SSc such as anti-nuclear factor, gammaglobulin, or immunological factors, anti-histone antibodies, increased collagen procollagen type III serum levels, and a decrease in anti-phospholipid antibodies are indicators of localized scleroderma [35-39].

Management

The following are certain management modalities [2,23,40-47].

Oral Submucous Fibrosis

Habit cessation involves the complete cessation of the habit or reducing the usage of areca nut (primary etiological agent). Topical treatment includes oral physiotherapy, local corticosteroids, intralesional injection of several enzymes such as hyaluronidase, colchicine with hyaluronidase, collagenase, antifibrotic cytokines, and other intercellular substances to decrease proliferation and abnormal deposition of collagen fibers. Systemic treatment includes micronutrient supplement therapy, antioxidants such as b-carotene, vitamins, lycopene, curcumins, Spirulina and aloe vera, immunomodulatory drugs, and systemic corticosteroids. Surgery with rehabilitative management includes submucosal resection and complete striping of fibrous bands, coronoidectomy, bilateral temporalis myotomy, followed by reconstruction with partial thickness skin grafts, buccal palatal graft, microvascular free radial forearm flaps, tongue flaps, and nasolabial flaps at the resected fibrotic site. Use of diode laser, KTP-532, CO2 laser, and ErCr: YSGG laser surgeries to promote healthy wound healing and reduce scar formation and trismus. Stem cell therapy through the intralesional injection of autologous bone marrow stem cells has shown a better prognosis by significantly increasing the mouth opening and reducing discomfort.

Scleroderma

Topical treatment includes topical corticosteroids, tacrolimus, phototherapy, and calcipotriol with PUVA (Psoralen ultraviolet A) cream, which has shown a better response rate in active diseases. Systemic treatment includes local scleroderma-systemic corticosteroids, methotrexate, D-penicillamine, oral vitamin D, psoralen-UVA (PUVA), phenytoin, cyclosporine, and interferon-alpha therapy. For diffuse scleroderma, the immunosuppressive drugs cyclophosphamide and mycophenolate mofetil are used. Calcium channel blockers were given for the management of CREST ((calcinosis, Raynaud phenomenon, esophageal dysmotility, sclerodactyly, and telangiectasia) syndrome in the early stages followed by Iloprost or bosentan for topical digit ulcers. In severe disease with extensive systemic organ involvement, oxygen therapy, anticoagulants, antibiotics, angiotensin-converting enzyme (ACE) inhibitors, diuretics, and corticosteroids were recommended to avoid multiorgan failure. For surgical treatment of severe cases with involvement of facial structures, palliative reconstruction surgery and physical therapy with ultraviolet radiation are beneficial modalities.

## Conclusions

OSMF possesses a significant rate of morbidity and mortality owing to its progressive masticatory difficulties with subsequent nutritional deficiencies and increased risk of the potential for malignant transformation. It has been well-established that dense fibrosis, decreased vascularity, persistent habit, chronic irritation, prolonged altered cytokine activity, and alkaloid secretions bring about surface epithelial changes and malignant transformation in OSMF. Localized scleroderma or morphea classically presents with benign and self-limited evolution when confined to the underlying tissue; however, the overall mortality and morbidity are high in systemic sclerosis, both in local and diffuse variants, representing a 27% to 72% death rate caused largely by cardiovascular and pulmonary involvement. Thus, appropriate assessment, determination of risk factors, early diagnosis, and intervention along with palliative care are essential to improve the overall prognosis, survival rate, and treatment outcomes and enhance the quality of life.
